# Physiotherapy-supervised mobilization and exercise following cardiac surgery: a national questionnaire survey in Sweden

**DOI:** 10.1186/1749-8090-5-67

**Published:** 2010-08-25

**Authors:** Elisabeth Westerdahl, Margareta Möller

**Affiliations:** 1 Department of Physiotherapy, Örebro University Hospital, 701 85 Örebro, Sweden; 2 Department of Cardiothoracic Surgery, Örebro University Hospital, 701 85 Örebro, Sweden; 3 Department of Medical Sciences, Clinical Physiology, University Hospital, 751 85 Uppsala, Sweden; 4 Centre for Health Care Sciences, Örebro University Hospital, Örebro County Council, Box 1324, 701 13 Örebro, Sweden; 5 School of Health and Medical Sciences, Örebro University, 701 82 Örebro, Sweden

## Abstract

**Background:**

Limited published data are available on how patients are mobilized and exercised during the postoperative hospital stay following cardiac surgery. The aim of this survey was to determine current practice of physiotherapy-supervised mobilization and exercise following cardiac surgery in Sweden.

**Methods:**

A prospective survey was carried out among physiotherapists treating adult cardiac surgery patients. A total population sample was identified and postal questionnaires were sent to the 33 physiotherapists currently working at the departments of thoracic surgery in Sweden. In total, 29 physiotherapists (response rate 88%) from eight hospitals completed the survey.

**Results:**

The majority (90%) of the physiotherapists offered preoperative information. The main rationale of physiotherapy treatment after cardiac surgery was to prevent and treat postoperative complications, improve pulmonary function and promote physical activity. In general, one to three treatment sessions were given by a physiotherapist on postoperative day 1 and one to two treatment sessions were given during postoperative days 2 and 3. During weekends, physiotherapy was given to a lesser degree (59% on Saturdays and 31% on Sundays to patients on postoperative day 1). No physiotherapy treatment was given in the evenings. The routine use of early mobilization and shoulder range of motion exercises was common during the first postoperative days, but the choice of exercises and duration of treatment varied. Patients were reminded to adhere to sternal precautions. There were great variations of instructions to the patients concerning weight bearing and exercises involving the sternotomy. All respondents considered physiotherapy necessary after cardiac surgery, but only half of them considered the physiotherapy treatment offered as optimal.

**Conclusions:**

The results of this survey show that there are small variations in physiotherapy-supervised mobilization and exercise following cardiac surgery in Sweden. However, the frequency and duration of exercises and recommendations for sternal precautions reinforced for the healing period differ between physiotherapists. This survey provides an initial insight into physiotherapy management in Sweden. Comparison with surveys in other countries is warranted to improve the physiotherapy management and postoperative recovery of the cardiac surgery patient.

## Background

Physiotherapy treatment is often prescribed to patients undergoing cardiac surgery, in order to prevent or diminish postoperative complications. The physiotherapy treatment during the hospital stay generally consists of early mobilization, range of motion exercises and breathing exercises. The value of postoperative chest physiotherapy has recently been established and accepted [1-4], but it is still unclear which treatment techniques are the most effective. In the literature a wide variety of treatments have been suggested. Many strategies and diverse therapies are applied postoperatively and these differ within and between countries. Early mobilization and physical activity is often the first choice of treatment, but evidence as to the optimal intensity, timing and choice of exercises is scarce.

There are only limited published data on how the cardiac surgery patient should be mobilized and exercised during the first postoperative period in hospital [4-7]. Physiotherapy management of patients undergoing coronary artery bypass graft (CABG) surgery [8] and thoracic surgery [9] has been examined in Australia and New Zealand. However, we found no such study performed in Europe.

This national survey was carried out to establish current clinical practice of physiotherapy-supervised exercise and mobilization, during the hospital stay, for patients having undergone cardiac surgery. A postal questionnaire survey was sent to all physiotherapists in Sweden working with this patient group, to determine which methods and treatments are used.

## Methods

A cross-sectional, descriptive study was carried out to examine the physiotherapy management and mobilization routines of cardiac surgery patients in Sweden. The study design was a national postal questionnaire survey sent to all 36 physiotherapists working at cardiothoracic centres in Sweden. The routine postoperative physiotherapy management of patients undergoing uncomplicated open-heart surgery, including CABG, mitral, aortic or tricuspid valve surgery, or a combination of these, was studied. Treatment of patients undergoing cardiac transplantation or other types of cardiac surgical procedures was not studied. The care of patients developing neurological symptoms, circulatory instability, prolonged intubation, or other conditions requiring individualized programmes was not considered. Physiotherapists who only treated patients undergoing other types of cardiac, pulmonary or thoracic surgery procedures were asked to return the questionnaire unanswered.

A total of 7,899 cardiac surgery operations were performed in Sweden during 2007, ranging from 310 to 1,635 across the eight different hospitals performing cardiac surgery. Median length of postoperative hospital stay was 9 days. The average physiotherapy staffing level for the Departments of Cardiothoracic Surgery was 3.2 (range 1.0 to 5.0) full-time equivalents.

### The questionnaire

Questions were asked about pre- and postoperative physiotherapy-supervised mobilization and exercise for hospital patients following cardiac surgery. The routine pre- and postoperative care of a hypothetical, "everyday patient" undergoing cardiac surgery was considered to determine the standard clinical practice. The questionnaire was developed for this specific study and constructed following a detailed review of the literature concerning physiotherapy treatment after cardiac surgery and previously developed questionnaires [8]. A range of both closed and open questions, about pre-operative and postoperative physiotherapy-supervised mobilization and exercise following cardiac surgery were included in the questionnaire. Results regarding specific breathing exercises are presented elsewhere. Respondents were also invited to make comments at the end of the survey.

A pilot test of the questionnaire was carried out prior to the main study. Six physiotherapists working at the Departments of Intensive Care, Cardiology or Lung Medicine at our hospital were asked to answer the questionnaire for comments on layout and contents. The questionnaire was then modified and some questions were rephrased. The questionnaire was translated from Swedish into English by one translator, and back-translated by another translator, to ensure correct formulation of the survey questions.

The study was performed during December 2007 and January 2008. All physiotherapists working at a Department of Cardiothoracic surgery in Sweden were sent a postal questionnaire. The questionnaire was addressed personally to the physiotherapists identified. The letter included a cover letter and prepaid, self-addressed response envelope. After 3 weeks, reminder letters with a copy of the questionnaire were sent to those physiotherapists who had not yet returned the questionnaire.

### Participants

Physiotherapists working at hospitals performing adult cardiac surgery in Sweden (Sahlgrenska University Hospital, Karlskrona Hospital, Linköping University Hospital, Lund University Hospital, Karolinska University Hospital, Umeå University Hospital, Uppsala University Hospital and Örebro University Hospital) were selected. The names and addresses of the physiotherapists had been identified and updated by the author, during a previous Swedish Thoracic Society meeting in October 2007. The names were double-checked via phone or mail by E.W. at each hospital just before the start of the study.

Before the questionnaire was sent, the head of the clinic at each selected cardiothoracic centre was contacted by e-mail, to get permission to carry out the study. Written informed consent was obtained from the head of the clinic granting permission for their physiotherapists to participate in the study.

The Regional Ethical Review Board were consulted in September 2007 regarding ethical approval, and advised that no formal ethical approval was required. The results from the questionnaire are confidential and no association between the results and a specific physiotherapist is possible.

### Statistical analysis

Descriptive statistics were used to analyse the results, and means, medians and ranges were calculated. SPSS 15.0 (SPSS Inc, Chicago, IL, USA) was used for the statistical analysis.

## Results

Of the 36 identified physiotherapists working at the departments of cardiothoracic surgery in December 2007, three could not be included in the study because of parental leave or because they were not working with the actual patient group. Responses were received from all hospitals to which the survey was sent. In total, 29 replies were received (giving an 88% response rate) out of the 33 questionnaires sent out. The physiotherapists were aged 41 ± 8 years and the mean work experience as physiotherapist at a department of cardiothoracic surgery was 6 ± 4 (range 1-16) years. Seventy-six per cent of respondents were women. Written physiotherapy guidelines or protocols for physiotherapy management of the cardiac surgery patient were available for 21 (72%) of the respondents.

All physiotherapists declared that they considered physiotherapy necessary after cardiac surgery and 55% considered the physiotherapy treatment offered at their department of cardiothoracic surgery optimal, while 31% found it not optimal and 14% said they did not know. Reasons for the treatment not being optimal were too many patients, lack of resources, shortness of care time, and increased care load.

The main purpose of physiotherapy following cardiac surgery was seen as preventing and treating postoperative complications, improving pulmonary function and promoting physical activity.

### Preoperative information

The majority (90%) of the physiotherapists offered preoperative information to all patients undergoing non-emergency cardiac surgery. The following topics were most frequently covered in the preoperative information: early mobilization (90%), post-sternotomy restrictions (90%), risk of postoperative pulmonary complications (90%), techniques for getting in and out of bed/the chair (80%), breathing exercises and coughing techniques (80%) and information about exercising the lower extremities (69%). The preoperative information was usually given to a group of patients by the physiotherapists (76%).

### Postoperative physiotherapy treatment

In total, 26 respondents answered that the physiotherapist automatically met all patients undergoing cardiac surgery while three said that they only met certain patients, with special needs, postoperatively. The physiotherapists reported that during weekdays they routinely treated patients on postoperative day 1 (90%), postoperative day 2 (93%), postoperative day 3 (69%) and postoperative days 4 and 5 (28%). The patients usually had between one and three treatment sessions with a physiotherapist on postoperative day 1, one to two treatment sessions on days 2 and 3, and typically one treatment on days 4 and 5. Physiotherapy treatment was never given during the evenings. On Saturdays, physiotherapy treatment was reported to be routinely given to patients on their first postoperative day by 59%, and only if needed by 41%, of the physiotherapists. The corresponding figures were 31%, and 14%, respectively, for Sundays, while 55% of physiotherapists never gave treatment on Sundays. On the second postoperative day, physiotherapy treatment on Saturdays was generally provided routinely by 17%, and only if needed by 83%, of the physiotherapists. If the second postoperative day fell on a Sunday, no routine physiotherapy was given, however, 48% of the physiotherapists responded that they would give physiotherapy treatment to patients if needed or advised from physicians.

### Mobility assessment

The following mobilization and exercise abilities were routinely assessed or recorded during physiotherapy treatment: mobility, getting in and out of bed/the chair (100%), circulation exercises for the lower extremities (72%); range of motion, shoulders and the upper extremities (62%); range of motion, thorax (59%); range of motion, cervical and thoracic spine (38%); functional activities of daily living (ADL) scores (21%); and exercise tolerance test, done by walking or bicycling (17%).

### Postoperative mobilization and exercises

Mobilization and exercises usually provided to the patients on the first postoperative days after surgery are presented in Tables 1 and 2. Instructions for range of motion exercises for the upper extremities and thorax were provided to the patients on postoperative day 1 by six physiotherapists, on postoperative day 2 by 22, and on postoperative day 3 by 25 of physiotherapists. How many times the patients were instructed to perform the exercises varied between one and three times a day during the hospital stay and once and twice a day after discharge. Postoperative group training for the patients during the hospital stay were provided by 62% of the physiotherapists. Physiotherapy-supervised stair climbing was practised postoperatively, according to 79% of the physiotherapists.

**Table 1:** Physiotherapy-supervised mobilization usually provided to cardiac surgery patients during the first postoperative days.

	POD 1	POD 2	POD 3	POD4
Mobilization				
sitting on edge of bed or in chair	97%	52%	48%	34%
standing	93%	55%	48%	34%
walking in the room	28%	79%	52%	34%
walking in the corridor	28%	66%	93%	41%
stair climbing	0%	0%	21%	38%
				
Positioning, side lying	24%	28%	10%	10%

Data shown as % of respondents (n = 29). POD = postoperative day.

**Table 2:** Physiotherapy-supervised exercises usually provided to cardiac surgery patients during the first postoperative days.

	POD 1	POD 2	POD 3	POD4
Thoracic/upper extremities ROM exercises				
Unilateral	3%	17%	34%	31%
Bilateral	10%	69%	76%	66%
				
Lower extremities ROM exercises	41%	31%	28%	24%
				
Relaxation techniques	14%	14%	7%	3%
Body awareness, posture exercises	3%	10%	14%	10%
Massage	0%	0%	0%	0%

Data shown as % of respondents (n = 29). POD = postoperative day; ROM = range of motion.

### Sternal precautions

Sternal precautions recommended for the healing period during the first postoperative weeks are presented in Table 3. Recommendations for how long after surgery the patients should avoid weight bearing varied between 7 and 12 weeks (mean 9 weeks). How much weight the patients were allowed to lift while the sternum was healing varied between 1 and 5 kg (median 2 kg, mean 2.5 kg).

**Table 3:** Sternal precautions recommended for the healing period during the first postoperative weeks after cardiac surgery.

Patients are not allowed to use:	n (%)
their arms to push up from a lying to a sitting position	5 (17%)
their arms to push up from sitting to standing	28 (97%)
their stomach muscles to raise themselves from a lying to a sitting position	12 (41%)
their arms and shoulders, using full active movement	1 (3%)
their arms and shoulders, using full active movement with 1-2 kg weights	12 (41%)
a rollator (rolling walker)	1 (3%)
a walker	0 (0%)
crutches	19 (66%)

Data shown as number (n) and as % of respondents (n = 29).

Instructions for moving in, and out of, bed were given to the patients using a "standard technique" by 90% of the physiotherapists. The most commonly described technique for getting out of bed was lying on the side, placing one or both hands in front of the body, leaning forward and pushing up to a sitting position.

### Postoperative information

Before discharge from the department of cardiothoracic surgery all physiotherapists provided information to the patients about physical activity, exercises and rehabilitation. Instructions to the patients to continue shoulder range of motion exercises after discharge from the hospital, were as well given by all physiotherapists. The time that patients were recommended to continue the exercise programme varied between 1 and 8 weeks.

## Discussion

This is the first survey to investigate and describe physiotherapy-supervised mobilization and exercise after cardiac surgery in Sweden. Most of the physiotherapists, in total 90%, declared that they routinely met all patients undergoing cardiac surgery, while 10% responded that they only treated certain patients, with special indications or special needs. The physiotherapy treatment was most frequently given on the first two postoperative days. On day 1 the patients usually received one to three treatment sessions by the physiotherapist, and on day 2, they were given one to two treatment sessions. The main purpose of physiotherapy after cardiac surgery was mostly seen as preventing and treating postoperative complications, improving pulmonary function and encouraging physical activity. Written local physiotherapy guidelines or protocols for physiotherapy management of cardiac surgery patients were available, according to 21 out of the 29 respondents.

Only one previous survey of physiotherapy management of patients undergoing cardiac surgery has been found, performed by Tucker et al. [8] in Australia and New Zealand. To our knowledge, our study is the first European survey describing physiotherapy treatment after cardiac surgery.

The clinical practice in Sweden and Australia and New Zealand seems to be similar in terms of the components of postoperative physiotherapy treatment, assessment of physiotherapy given to all patients (89%), and mobilization and breathing exercises, as described by Tucker et al. [8]. However, the study was carried out in 1996, so we do not know how their clinical routines and practice come across and may differ today. More recently physiotherapy management after thoracic surgery was described in a survey study by Reeve et al. [9], however the physiotherapy treatment following thoracotomy cannot be compared to treatment after cardiac surgery.

In total, 29 replies were received out of the 33 questionnaires sent out. Since the questionnaires were comprehensive the response rate of 88% can be considered high. A high response rate is important and various strategies were used to improve the response rate. Comprehensible instructions were given, the questionnaires were printed on coloured paper; stamped, addressed envelopes were included with the questionnaires and reminders were sent out where the questionnaires had not been returned.

Access to a list of all physiotherapists working in departments of cardiothoracic surgery as well as personal contacts with physiotherapists at all departments ensured that all relevant physiotherapists were included in the survey. The study of a total population sample and the high response rate gives the study good external validity. It is likely that the results of this survey reflect current practice in Sweden, even if some important questions may have been overlooked and the exact description of the actual clinical practice, in observational studies, is warranted in the future.

An intrinsic selection bias in questionnaire studies is a risk if only the most motivated physiotherapists respond. Since only four physiotherapists failed to answer, we found this risk of bias fairly low. Because no nationally developed questionnaire for this purpose existed, the authors designed the questionnaire. To improve the content validity of the survey, information from earlier questionnaires used in similar studies [8,9] as well as pilot testing was used to construct the questionnaire. Despite these limitations we believe that the results from this survey provides a good overview of the physiotherapy treatment given to cardiac surgery patients.

The majority (90%) of the physiotherapists offered preoperative information to all patients undergoing non-emergency cardiac surgery, which is similar (94%) to the routines in Australia and New Zealand described by Tucker at al. [8]. The educational content of the preoperative information was similar, with early mobilization, post-sternotomy recovery and postoperative pulmonary function being the topics most covered.

Treatment was generally less comprehensive during weekends. Routine physiotherapy for patients on their first postoperative day was given more often on Saturdays (59%) than on Sundays (31%). For patients on their second postoperative day, no routine physiotherapy was given on Sundays, except where needed, as reported by half of the physiotherapists. These results indicates that there is a discrepancy in treatment of patients depending on which weekday they are operated on in Sweden. By comparison, in Australia and New Zealand during the 1990's, evening services were provided as required in 71% of hospitals, while in Sweden no evening physiotherapy treatment is available at all.

In the late 1960 s, patients would spend at least 3 weeks resting in bed after cardiac surgery. Since then the practice of postoperative physiotherapy has changed in response to advances in medical and surgical knowledge [10]. Today there is an agreement as to the value of early mobilization and positioning after cardiac surgery [11-13], despite the risk of postoperative cardiac dysfunction [6,14]. Almost all physiotherapists in our study mobilized their patients with regard to sitting and standing on postoperative day 1. Invasive cardiovascular monitoring is common in the early postoperative period and affects the ability to walk a longer distance from the bed because of the equipment.

Of course, it is the individual strength and cardiovascular status of the patient that decides the level and intensity of mobilization. In this study the average mobilization routines performed by a physiotherapist of a hypothetical "everyday" patient was determined. The actual mobilization of individual patients has not been the focus of the present study. Despite the frequent use of early mobilization, the benefit of mobilization in preventing postoperative complications has not been studied in the cardiac surgery patient. Studies' investigating different levels of mobilization during the hospital stay are lacking. In a recent follow-up of CABG patients, work capacity, and participation in household work were described as predictors of continuation at work after the surgery [15]. The authors encouraged medical personnel to activate the cardiac surgery patient to undertake household work and all kinds of physical activities [15].

By contrast, positioning to a side-lying was used only by approximately 25% of the physiotherapists during the first postoperative days, despite the fact that positive effects of side lying on lung volumes [12] and oxygenation [16] have been described. Patients possibly experience increased pain and discomfor in this position, which may be an explanation for the low frequency of use.

All physiotherapists provided information about physical activity, exercises and rehabilitation to patients after discharge from the hospital. The content of the information would be interesting to study further, as could recommendations and regimens from cardiac surgeons, anaesthesiologists and cardiologists.

Shoulder range of motion exercises are today a common form of therapy intended to improve ventilation, preserve thorax mobility and ease sternal circulation and healing [17], even though the efficacy of shoulder range of motion exercises has been questioned [5].

Instructions in range of motion exercises for the upper extremities and thorax were mostly started on postoperative days 2 and 3. Only six of the physiotherapists started these exercises on the first postoperative day. It is currently not known how these exercises should be performed. In a study of patients with chronic sternal instability, by El-Ansary et al. [18], it was shown that bilateral upper limb movements were significantly less associated with sternal pain compared with unilateral movements. In the present survey, mostly bilateral upper extremity exercises (69%) were prescribed, rather than unilateral range of motion exercises. How many times the patients were instructed to perform the exercises varied between one and three times a day. Shoulder range of motion exercises, to be continued after discharge, were given by all physiotherapists. Recommendations for continuing the exercise programme varied between 1 and 8 weeks, however.

Recommendations for sternal precautions during the first postoperative weeks differed, which may reflect differences between recommendations from thoracic surgeons and hospital policy. Diverse instructions were given regarding restrictions of using of arms to push up from a lying to a sitting position, using the stomach muscles and also using crutches. However, almost all of the physiotherapists allowed the patients to use their arms to push up from sitting to standing position, move their arms and shoulders in full active movement, and use rolling walkers and walkers. Instructions for moving in and out of bed were given to the patients using a "standard technique" by 90% of the physiotherapists. The most commonly described technique for getting out of bed was from side lying, placing one or both hands in front of the body, leaning forward and pushing up to a sitting position.

Many activities are discouraged after cardiac surgery, such as weight carrying and exercises involving the pectoralis major. Few studies have been published evaluating which activities and exercises negatively affect the sternal incision [18-20]. The recommendation for how long after surgery the patients should avoid weight bearing and certain other activities, differs with a range of 7 to12 weeks. Likewise, how much weight patients are allowed to lift while the sternum is healing differs between 1 and 5 kg. It has been suggested that current activity guidelines for CABG patients are too restrictive [21]; however, considering that postoperative sternal instability is a serious complication with increased risk of mortality, the importance of correct instructions for sternal precautions is essential, especially in risk patients [22]. More scientific knowledge of risk factors and risk behaviours for sternum instability is needed. This would provide further possibilities to individualize the postoperative recommendations to the patients.

All physiotherapists in the present study considered physiotherapy necessary after cardiac surgery, although one-third considered the physiotherapy treatment offered not optimal. The main reason mentioned was lack of time.

A national Swedish guideline for physiotherapy treatment for patients undergoing major surgery is currently under development, but was not available during the study period. In spite of this, the physiotherapy management given in the different departments, by different physiotherapists, was fairly similar. An explanation for this may be the yearly national meetings for physiotherapists in the cardiovascular field. This survey provides information that may be useful in research as well as development and implementation of clinical practice guidelines in physiotherapy. It is also very important to widen this knowledge and formulate internationally accepted guidelines for cardiac surgery patients.

## Conclusions

This survey provides initial insight into physiotherapy management in Sweden.

The results of the survey indicate that there are only small variations in physiotherapy-supervised exercise and mobilization following cardiac surgery in Sweden. The routine use of early mobilization and upper extremity exercises is common during the first postoperative days, although the frequency and duration of exercises vary. The study shows a discrepancy in physiotherapy treatment accessibility to patients, depending on the weekday they are operated on. Sternal precautions are given routinely and cardiac surgery patients receive standardized instructions for getting into and out of bed. However, the advice given for the healing period differs between physiotherapists. Further research and development of high-quality clinical guidelines as well as comparison with routines in other countries is needed to confidently promote the postoperative recovery of the cardiac surgery patient.

## Competing interests

The authors declare that they have no competing interests.

## Authors' contributions

EW designed the study and questionnaire, performed the statistical analysis and wrote the manuscript. MM contributed to the design of the questionnaire and helped to draft the final manuscript. Both authors read and approved the final manuscript.
